# Creation of Polymeric Organosilicon Layers on the Surface of Pipeline Steel for Inhibition of Stress Corrosion Cracking

**DOI:** 10.3390/polym18111357

**Published:** 2026-05-29

**Authors:** Liudmila B. Maksaeva, Vasiliy E. Ignatenko, Alevtina A. Rybkina, Tatiana A. Yurasova, Maxim A. Petrunin

**Affiliations:** Frumkin Institute of Physical Chemistry and Electrochemistry Russian Academy of Sciences, 31-4, Leninsky pr., 119071 Moscow, Russia; lmaksaeva@mail.ru (L.B.M.); basil148@mail.ru (V.E.I.); aa_rybkina@mail.ru (A.A.R.); tatal111@yandex.ru (T.A.Y.)

**Keywords:** corrosion-mechanical testing, stress corrosion cracking, organosilanes, siloxane surface layers, inhibition of stress corrosion cracking (SCC)

## Abstract

The article deals with the study of stress corrosion cracking (SCC) of X70 steel using corrosion-mechanical testing that simulates the operating conditions of underground pipelines. The tests were carried out under cyclic four-point bending at stresses close to the yield point, in electrolytes with various hydrogen charging capacities. The following model environments were used: NS4 solution and citrate buffer (pH 5.5). Hydrogen charging was controlled by the addition of thiourea and by varying the potential. It was shown that microcracks initiated at corrosion defects (pits) and then emerged at the surface to form narrow cracks. The incubation period depends on the environment: under corrosive conditions it is approximately two times shorter than in the air. The size and nature of stress concentrators play a significant role: natural pits (~hundreds of μm) lead to crack formation within 24–28 days, whereas artificial holes (0.6–1 mm) lead to crack formation within 5–7 days. The effect of hydrogen was established: the acceleration is insignificant under moderate hydrogen charging, whereas the incubation period decreases sharply at high hydrogen charging. Critical hydrogen concentrations where its effect becomes significant were determined. Methods for inhibiting stress corrosion cracking by means of organosilicon films (vinyl- and aminosilanes, as well as their mixtures with inhibitors—benzotriazole and amines) were considered. The most effective composition is vinylsilane + benzotriazole: the time to crack initiation increases from 5 to 36 days, and the crack growth rate decreases.

## 1. Introduction

Steel pipelines serve as the primary means of transporting hydrocarbons due to their economic efficiency, reliability, and safety [1,2]. Low-alloy steel is widely used as the main structural material for oil and gas pipelines [3,4] but, despite its operational advantages, it exhibits low corrosion resistance. Analysis of operational data [5,6,7] shows that corrosion remains one of the key causes of damage in underground pipelines and can lead to significant economic losses [8,9,10,11], environmental damage [12,13], and other losses and risks of accidents. One of the most dangerous forms of corrosion damage is stress corrosion cracking (SCC) [14,15], which occurs under the combined action of tensile stresses and an aggressive environment. The development of SCC can be sudden and catastrophic, leading to pipeline ruptures, leaks and spills of petroleum products, severe environmental damage, and human losses. Despite a decrease in the frequency of such accidents in recent decades, cases of pipeline ruptures due to SCC continue to occur [12,16].

The mechanism of initiation and propagation of SCC is determined by multiple factors. In particular, it depends on the chemical composition of the environment. According to the literature data [17,18], the following causes of SCC are recognized: in relatively alkaline environments (pH ~ 8–11), anodic dissolution of the crack tip predominantly occurs, whereas in near-neutral environments (pH ~ 5.5–7.5), both the mechanism of anodic dissolution of the crack tip and the steel hydrogen embrittlement mechanism can operate.

To date, several mechanisms for the effect of hydrogen on the crack initiation and propagation in steel have been proposed. Hydrogen adsorbed by the metal promotes the initiation of the pitting corrosion in the presence of the corrosive ions due to the disruption of the passive oxide film [19]. It was found [20] that pitting develops more rapidly on hydrogen-charged steel in chloride and chloride-bicarbonate solutions. Moreover, absorbed hydrogen increases the probability that small metastable pits will transform into stable ones due to an increase in the rate of steel dissolution and a decrease in the rate of surface repassivation [21]. The resulting pits are potential sites for crack initiation due to local concentration of mechanical stress and localized plastic deformation of the metal in the pit region [22,23].

In a number of studies [24,25], the models were developed describing both pit growth and conditions of its transformation into a microcrack, taking into account the geometry (depth and diameter) of the pit, the density of surface coverage by pits, and the magnitude and mode of mechanical loading of the metal. Moreover, it was shown [25] that the fastest crack formation occurred under local stress in the pit region, exceeding the ultimate tensile strength under the low-cycle loading conditions of the steel. However, as a rule, the effect of cathodic hydrogen evolution and hydrogen penetration into the metal on the process of pit-to-microcrack transition has not been considered by the authors.

A microcrack initiated from a pit develops under cyclic loading conditions. The mechanism of crack growth is most commonly attributed [26,27] to hydrogen-enhanced localized plasticity (HELP), which, as well as a number of alternative mechanisms, implies the acceleration of crack growth with increasing hydrogen content in the steel above a certain critical level. Several other partially competing models describing the mechanism of hydrogen-induced cracking of metal are proposed in the literature, namely: HEDE (hydrogen-enhanced decohesion), AIDE (adsorption-induced dislocation emission) and, for hydride-forming metals, DHC (delayed hydride cracking). In studies on magnesium alloys and steels, these models are used to explain how atomic or adsorbed hydrogen facilitates dislocation motion, reduces boundary cohesion, or stimulates local crack opening [28]. It should be noted that, despite the large number of studies, there is no unified understanding of the factors determining the initiation and propagation of stress corrosion cracking in large-scale structures. Furthermore, despite the risks associated with this type of damage, there is a lack of information in the literature regarding methods to mitigate it. The literature on this subject notes that: hydrogen absorption inhibitor must be involved in the earliest stages of the process—the adsorption of electrolytic hydrogen on the metal surface to form H_ads, its retention at active sites, and its incorporation into the metal matrix, i.e., hydrogen absorption and the transition from H_ads to H_abs. If an inhibitor reduces only general corrosion, it may prove to be a poor inhibitor of hydrogen-induced cracking. Therefore, one should not draw conclusions about the effectiveness of SCC inhibition based solely on the high efficiency of uniform corrosion inhibition [29]. The literature suggests the following approaches to inhibiting hydrogen cracking: (a) the use of film-forming organic corrosion inhibitors capable of adsorbing on the active centers of steel, reducing the area of cathode sites and blocking the penetration of atomic hydrogen into the volume of the metal. Examples of such inhibitors include the action of benzotriazole (BTA) on X65 steel during cathodic polarization and imidazole derivatives during steel etching [29,30]; (b) the use of synergistic organo-inorganic systems capable of combining an adsorbent organic film with an oxide/passivating layer, shielding film defects and hindering access to the metal. Examples include mixtures of BTA with Na_2_Mo_4_; BTA with NaNO_2_ and BTA with Na_2_Mo_4_, with each mixture considered as a separate inhibiting composition acting according to its own mechanism [31]; (c) the use of cationic surfactants and ion pairs that facilitate the delivery and retention of the organic anion on the negatively charged/polarized surface and provide more complete coverage of the surface with the inhibitory layer. Examples of this approach in the literature include the action of mixtures of cetyltrimethylammonium cation (CTA) with trans-4-hydroxycinnamate (40Hcinn-)—a mixture: [CTA-40Hcinn] and a mixture of cetyltrimethylammonium cation with trans-4-ethoxycinnamate (4EtOcinn-)—a mixture: [CTA-4EtOcinn-] [32]; (d) in the case of hydrogen embrittlement in hydrogen pipelines—the addition of small amounts of certain gases such as O_2_, CO and SO_2_ can reduce hydrogen embrittlement in high-pressure hydrogen pipelines, since these gases are able to compete with H_2_ for adsorption at surface sites and can influence the course of surface hydrogen dissociation/adsorption reactions [33]; (e) creation of barrier coatings and passivating layers with high barrier properties, such as oxide/molybdate films, and metal or polymer barrier layers. However, the same literature notes [30,31,32,33] that almost all of these approaches have drawbacks, often limiting their widespread use. Thus, to inhibit hydrogen penetration into metal, the presence of inhibitor molecules in solution is insufficient, and even the adsorption of these molecules on the metal surface is insufficient. Formation of an inhibitor film on the steel is necessary [30], and it is desirable for the film to have a minimum of defects and maintain its integrity throughout the entire testing (operation) period. Furthermore, the film must be preserved under deformation, in cracks, and under a layer of corrosion products. Another drawback of some of the proposed inhibitor approaches is that, while they have demonstrated good results in laboratory studies, they require practical verification, such as benchtop and field testing.

In recent years, organosilanes—compounds with the general formula R_n_Si(OR′)_4−n_ (where R is an organic radical of the molecule and OR′ is a readily hydrolyzing group)—have attracted particular interest of researchers due to the ability of these compounds to form strong bonds with metal surfaces [34,35], and after condensation of neighboring adsorbed molecules, generate a surface polymeric film [35]. Previously, they were predominantly used to enhance interfacial interactions in composite materials [34]; however, recent studies consider organosilanes as promising corrosion inhibitors [35], including stress corrosion cracking inhibitors [36]. Thus, organosilanes can be considered as film-forming corrosion inhibitors, and the formed polymer (siloxane) film is stable and remains on the metal after corrosion tests under conditions that provoke intensive dissolution of the metal (sodium chloride solution) [37]. In addition, it was shown both in laboratory tests and during bench tests at the testing site of the polymer coating manufacturer that the introduction of organosilanes into a bitumen-polymer coating for main gas pipelines ensures the inhibition of SCC of pipe steel without deteriorating the adhesion of the coating to the metal [36]. And it was previously shown [38] that the siloxane film on the surface reduces the intensity of the cathodic reaction of hydrogen discharge and inhibits the penetration of hydrogen into the steel. All of the above allows us to expect that organosilanes will form polymer layers on the steel surface capable of inhibiting SCC of steel. The scientific novelty of this work will consist in the fact that for the first time, using a direct research method, the possibility of inhibiting both the initiation and development of a corrosion crack will be demonstrated. The goal of this work was to study the initial stages of SCC in X70 pipeline steel, to elucidate the effect of key factors, including different concentrations of hydrogen in the metal, on the initiation and propagation of stress corrosion cracking in pipeline steel, and to estimate the potential for inhibiting SCC processes by modifying the metal surface with formulations based on organosilanes.

## 2. Materials and Methods

**Figure 1:**
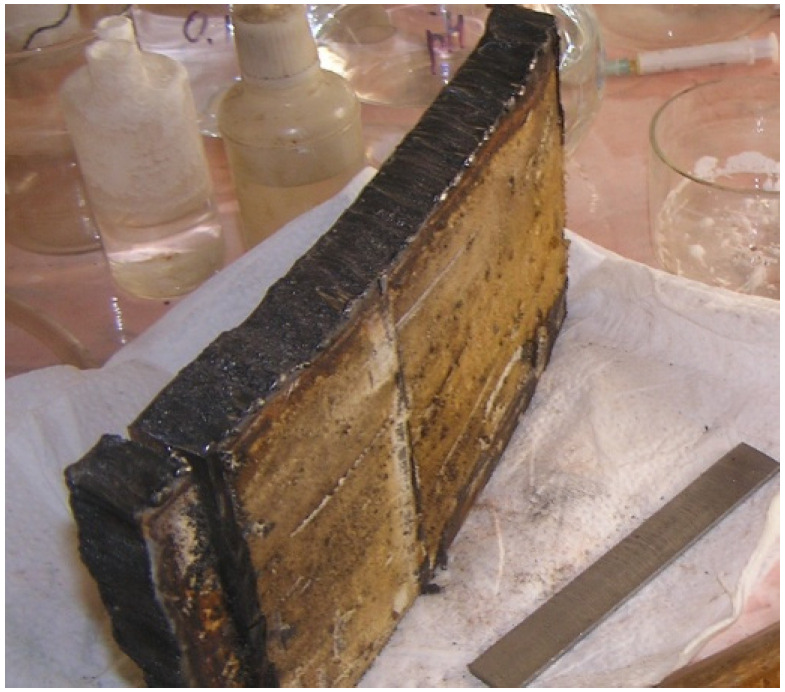
A pipe wall fragment and a beam specimen cut for testing.

Steel specimens of X70 strength grade were tested. The specimens were prepared from a section of a decommissioned pipe (Figure 1) from a Russian main gas pipeline. Pipes manufactured by the Khartsyzsk Pipe Plant, Khartsyzsk city, Donetsk region, Russia (KhTZ), and by Mannesmann AG, Duisburg, Germany (“Mannesmann”), were selected for the specimens. The chemical compositions of the metal specimens are presented in Table 1.

**Figure 2:**
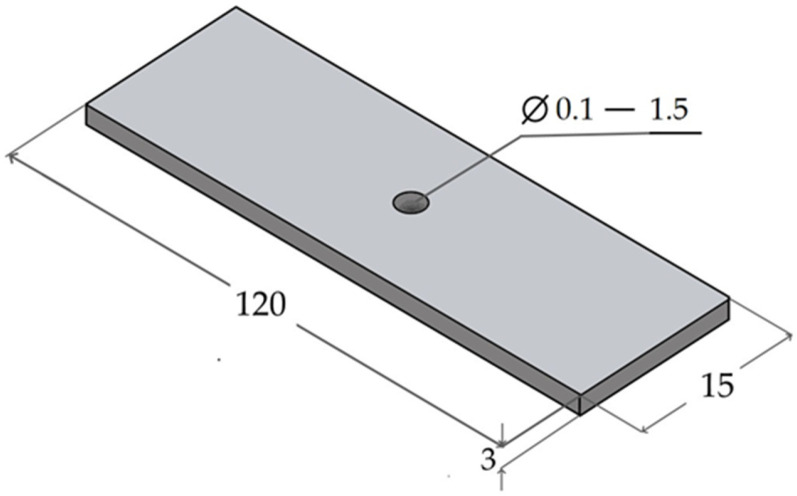
Specimen for corrosion-mechanical testing. Four-point bending method.

Beam specimens with dimensions of 130 mm in length, 15 mm in width, and 3 mm in thickness were used (Figure 2).

**Figure 3:**
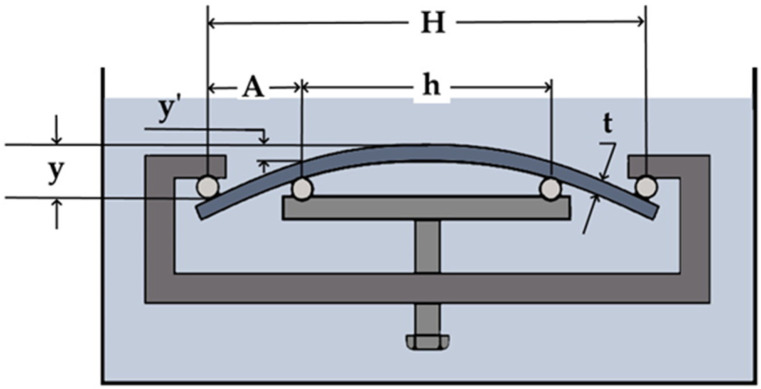
Scheme of the specimen loaded in four points [39,40]. H—the distance between the outer supports; y—the adjustable maximum deflection of the specimen [39]; h—the distance between the inner supports; t—the specimen thickness; y′—deflection between the inner supports; A—the distance between the outer and inner supports. (the recommended ratio is A = H/4 [39]).

Corrosion-mechanical testing of the pipeline steel specimens was carried out in a setup providing cyclic mechanical loading in accordance with the four-point bending scheme (Figure 3) [39,40] in a corrosive environment. Cyclic loading (0.15 Hz) was applied using a rotating eccentric mounted on the shaft of an electric motor with a gearbox.

The tensile stress on the specimen outer surface (σ, N/m^2^) was calculated using Equation (1) in accordance with GOST 9.901.2–89, which is equivalent to ISO 7539-2:1989 [39]:

(1) $$\sigma =\frac{12Ety}{3H^{2}-4A^{2}},$$

where *E* is the elastic modulus of steel (*E* = 2.06 × 10^5^ N/m^2^); *t* is the thickness of the specimen; *y* is the maximum deflection between the outer supports; *H* is the distance between the outer supports; and *A* is the distance between the outer and inner supports.

The deflection of the specimen was set in such a way that the maximum tensile stress on the outer surface of the specimen was 480 MPa, which matches the yield strength of the steel under study. For the given geometry of the specimen and given test scheme, the deflection was 1.8 mm. The stress ratio, i.e., the ratio of the minimum load to the maximum load, amounted to zero. Pit simulators in the form of drilled blind holes with diameters ranging from 0.1 to 1.5 mm and depths approximately equal to their radii were made on the specimens. The simulators had a shape close to a hemisphere (aspect ratio 2:1). In some series of experiments, the pit simulators were made by anodic etching of a specimen in an alkaline solution [41] (“natural pits”) instead of drilling holes. For this purpose, the specimen surface was coated with a lacquer, in which punctures were made with a needle to enable anodic etching. The resulting pits with a diameter of about 300 μm also had a hemispherical shape (aspect ratio 2:1).

The following test solutions (environments) were used:

(a) Citrate buffer solution (CBS), pH 5.5 (0.015 M citric acid + 0.085 M sodium citrate);
(b) CBS with the addition of thiourea as a hydrogen charging promoter, at a concentration of up to 10 mM to increase hydrogen penetration into the steel;
(c) Artificial test solution NS_4_ simulating a natural electrolyte that is accumulated under an isolation coating peeled off from the pipe [42] (Table 2), based on a borate buffer solution (BBS), pH 6.7 (0.4 M H_3_BO_3_ + 5.5 mM Na_2_B_4_O_7_·10H_2_O).

In each experiment, three samples were tested in parallel under identical conditions.

To evaluate the possibility of inhibiting stress corrosion cracking, organosilicon polymer layers were applied to the surface of the steel specimens. For this purpose, the specimen surfaces were modified with solutions of organosilicon solutions containing organosilanes and organic corrosion inhibitors. The compositions of the solutions are presented in Table 3.

The modification was carried out by immersing the specimens for 10 min in the modifying solution; after that, the specimen was immersed for 1 min in a solvent (water, or in the case of BTA, a water-alcohol mixture) to remove excess unreacted modifier and then air-dried for 120 min at room temperature.

To determine the amount of organosilane on the surface before and after modification, the specimens were weighed with an AF-R220CE analytical balance (Shinko Denshi Co., Ltd., Tokyo city, Japan). The measurement error of the sample mass is 0.1 mg. The layer thickness *h* (μm) was calculated from the mass difference using Equation (2) assuming uniform distribution of the layer over the surface:

(2) $$h(\mu m)=10,000\frac{\Delta m}{\rho S},$$

where *Δm* is the change in the mass of the specimen after modification (g), *S* is the area of the “working” surface of the specimen (cm^2^), and *ρ* is the density of the organosilane (g/cm^3^).

Tabulated values of organosilane density were used. For the mixtures, the arithmetic mean of the mixture components’ densities was used.

The amount of hydrogen absorbed by the steel was evaluated by the electrochemical desorption method proposed in [43]. Procedure details are described in [44,45]. Membranes were fabricated from X70 pipeline steel; their thickness was 0.5 mm, and the surface area in contact with the solutions was 4.25 cm^2^. The detection side of the membrane was coated with a palladium layer to prevent passivation in alkaline solution. The procedure for palladium deposition on the detection side of the membrane and the preparation of the membrane surfaces prior to the measurements are described in Ref. [45]. The detection compartment of the cell was filled with 0.1 M NaOH solution, in which the membrane was polarized at a potential of 0.25 V. The hydrogen flux (*i_p_*) emerging from the detection side of the membrane was determined as the difference between the ionization current (*i*) and the background current (*i_bg_*) (Equation (3)):

(3) $$i_{p}=i-i_{bg},$$

The steady-state hydrogen permeation currents (*i*_*p*,*st*_) were used to determine the hydrogen concentration within the steel membrane. The maximum hydrogen concentration under steady-state conditions is created in the subsurface layer of the metal on the working side of the membrane. The near-surface hydrogen concentration in steel (*C*_*H*,*S*_, ppmw) was calculated using Equation (4):

(4) $$C_{H,S}=\frac{i_{p,st}L}{F\rho D_{H}},$$

where *ρ* is the density of steel, *F* is the Faraday constant, *L* is the membrane thickness, and *D_H_* is the diffusion coefficient of hydrogen in steel.

To calculate *C*_*H*,*S*_, it is necessary to know the hydrogen diffusion coefficient in steel (*D_H_*). There is a large scatter of data in the literature concerning hydrogen diffusion coefficients; the values may differ by orders of magnitude. As a rule, *D_H_* is determined from the dependence of *i_p_* on time and strongly depends not only on the composition and structure of the steel and the stress level in the metal, but also on the degree of filling of hydrogen traps, which varies depending on the hydrogen flux through the membrane. As a result, *D_H_* depends on the composition of the environment, electrode potential, and the preparation of the membrane surface. In the present work, a *D_H_* value of 1 × 10^−6^ cm^2^/s was used for X70 pipeline steel in the neutral medium and 2.09 × 10^−6^ cm^2^/s in the acidic medium [46].

Table 4 presents the values of hydrogen penetration current and the corresponding calculated *C*_*H*,*S*_ values obtained in the NS4 + borate buffer solution (pH 6.7) and citrate buffer solution (pH 5.5), with and without the addition of hydrogen charging promoters.

Optical microscopy of the modified surface of steel specimens was performed using a 136 MMR-34 inverted metallographic microscope (LOMO MA LLC, Saint Petersburg city, Russia) equipped with a UI–1460LE–C–HQ digital video camera (IDS Imaging Development Systems GmbH, Obersulm city, Germany) mounted on the eyepiece. The camera resolution was 2048 × 1536 pixels. The data from the camera were transferred to a personal computer and processed using uEye Cockpit 4.96.1 software.

## 3. Results and Discussion

During corrosion–mechanical testing by the four-point bending method, it was found that microcracks appear on the surface of the tested steel under cyclic loading both in the presence of a corrosive environment and in the air. In the presence of the corrosive environment, the crack initiation occurs faster than in the air, i.e., in the absence of the environment (Table 4).

**Figure 4:**
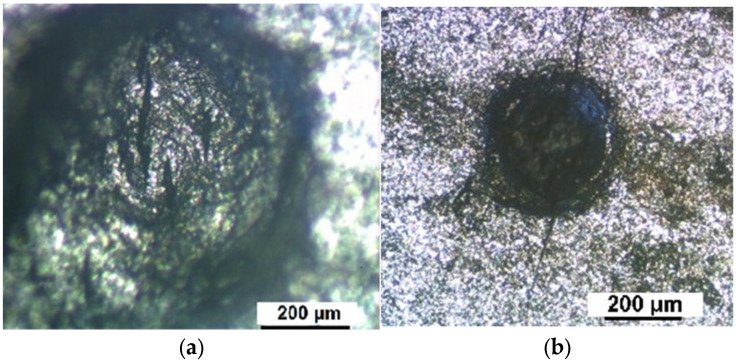
Formation of microcracks at the bottom of pits on the surface of KhTZ pipeline steel (**a**) and crack propagation along the surface (**b**). Four-point bending method. Solution: NS4 + borate buffer (pH 6.7). Corrosion potential.

Figure 4 shows microcracks formed from pits. It can be observed that the microcracks initiate at the bottom of the pits, then extend onto the main surface of the steel, and propagate rapidly, becoming narrow with a high aspect ratio.

The initiation time for microcracks from “natural” pits measuring 300 μm under the experimental conditions was 24 and 28 days for KhTZ and Mannesmann pipes, respectively. The subsequent crack growth rate after initiation, both in the corrosive environment and in the air, was approximately 7–13 μm/day for Mannesmann pipes and 10–15 μm/day for KhTZ pipes.

**Figure 5:**
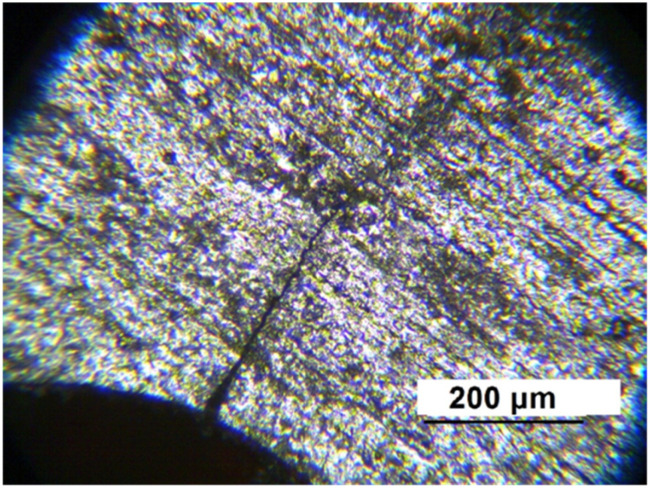
Formation of microcracks from the hole with the diameter of 1000 μm on the surface of KhTZ pipeline steel. Four-point bending method. Solution: NS4 + borate buffer (pH 6.7). Corrosion potential.

When testing the specimens with drilled holes as artificial defect initiators, crack formation occurred earlier, namely, after 5–7 days of testing, from the edges of holes with diameters of 600–1000 μm (Figure 5). The subsequent crack growth after initiation, both in the corrosive environment and in the air, occurred at the same rate as in the case of “natural” pits: 7–13 μm/day for Mannesmann pipes and 10–15 μm/day for KhTZ pipes.

The crack mouth width after 5 days of corrosion-mechanical testing was less than 5 μm and the crack length was 60 μm.

Upon further testing of the specimens with the formed crack in the NS4 solution, crack growth continued. In fact, after 15 days of testing, the crack mouth width reached 10 μm, while the crack length ranged from 50 to 100 μm. After 24 days of testing, the crack dimensions increased significantly: the crack mouth width was up to 20 μm and the crack length was up to 300 μm.

It would undoubtedly be useful to characterize the structure of the metal surface area where cracking occurs, but it is difficult to do this experimentally, since we are talking not so much about the area of the external corroding zone, but about the local corrosion-electrochemical-mechanical system in the area of the crack. Characterization of such a system is difficult because it must be carried out “in situ” during the process of crack initiation and growth, and this is not always possible. Therefore, when describing the possible structure of the active surface area on which a crack originates and develops, we had to rely on literary data. It should be noted that in the case of SCC. the actively corroding area is usually concentrated at the tip of the crack. The bulk medium outside the crack and the solution/electrolyte inside are different environments: a closed, poorly mixed “occluded” cell develops within the crack, where the potential and concentration of electrolyte ions, including pH (i.e., the concentration of the depolarizer responsible for the cathodic process), change. In [47] directly stated that the conditions at the crack tip are not described by the conditions of the bulk solution, and crack growth is determined by local chemical, electrochemical, and stress conditions at the tip.

The surface area where metal cracking occurs includes several sections (zones) (table), each of which has its own characteristics and a certain influence on the crack formation process as a whole:

(a) External surface and crack mouth. On the external surface, the metal may appear relatively undamaged: SCC often manifests itself as thin, branched cracks rather than as a uniform loss of metal. Morphologically, cracks can be intercrystalline—along grain boundaries, or in near-neutral environments. Transcrystalline [48]—through grains, or mixed: this depends on the material-environment pair [49,50];
(b) The crack walls are not necessarily the primary site of dissolution. In a typical scheme for aqueous media, the main anodic reaction is concentrated near the crack tip, while cathodic reactions occur more frequently at the crack mouth and on the walls closest to it. Oxygen inside the crack is quickly consumed, and its delivery to the tip is limited. Therefore, a more negative potential forms near the tip. Which becomes a local anode [47];
(c) The crack tip is the active anodic zone. This is the main “corroding region” in the narrow sense. Here, under the influence of high stress concentration and localized plastic deformation, the passive film is disrupted, fresh metal is exposed, and anodic dissolution and subsequent repassivation occur. In film rupture/slip-dissolution models, the passive film at the crack tip periodically breaks down, the metal dissolves, and then the surface repassivates; repetition of this cycle maintains crack propagation [51,52];
(d) Solution at the crack tip. The chemistry of the solution at the tip has distinct layers, and its composition differs significantly from that of the bulk solution “above the crack.” Due to anodic dissolution, metal ions accumulate at the tip; their hydrolysis leads to acidification of the local environment inside the crack. In a number of experiments for Al and Fe alloys, the pH at the tip of SCC cracks was around 3–4, even with a more neutral bulk solution; for steels in 3.5% NaCI. pH values of around 3.7 are given. However, this is not observed in every case of crack development: in individual cases, exceptions to this rule are possible [47]. Some systems provide exceptions, for example, with cathodic polarization, the pH of the solution at the crack tip can increase.
(e) Solution inside the crack. During crack development, a change in the ionic concentration of the solution and “locking” of the medium are observed. A gradient of the electrolyte composition is realized inside the crack. To maintain electroneutrality, anions migrate to the crack tip, including aggressive Cl^−^ anions; therefore, at the crack tip, an increased concentration of simultaneously dissolving metal cations, hydroxonium ions formed as a result of hydrolysis of corrosion products, and anions from the external solution is observed. The review [47] presents data that at the tip of SCC cracks in 7050 alloys, the Cl^−^ concentration could be approximately an order of magnitude higher than in the bulk solution:
(f) Pre-crack tip zone. Immediately preceding the crack tip is the process zone: high stress concentration, localized plastic deformation, dislocations, hydrogen traps, grain boundaries, precipitates, and alloying element-depleted zones. This is where fracture mechanics and electrochemistry meet. Work on 7xxx Al alloys at the atomic level has shown that hydrogen segregates to dislocation structures and grain boundaries ahead of a propagating SCC crack: Mg-rich amorphous hydroxide has also been detected on the corroded crack surface [53];
(g) The zone of cathodic discharge of hydronium ions. During SCC, anodic dissolution and hydrogen embrittlement often act together. For pipe steels, it has been shown that anodic dissolution is active inside the crack, and the cathodic discharge of protons forms hydrogen, which enters the steel at the crack tip: inside the crack, the environment becomes more acidic, and the rates of anodic and cathodic reactions increase [54]. Another study on pipe steels in near-neutral solutions indicated that hydrogen diffuses into the steel around the crack tip, accelerates dissolution, and increases the intensity of SCC [49];
(h) Initiation sites: pitting and local defects. Before the formation of a stable SCC crack, a local defect often occurs—pitting, crevice corrosion, passive film damage, inclusion, grain boundary, weld zone, or residual stress zone. A study of AISI 4135 steel in the offshore tidal zone noted that under tensile stress, cracks tend to initiate and propagate from corrosion pits due to stress concentration: acidity within the pits and local anodic dissolution further facilitate hydrogen penetration [54].

In general, the structure of the corroding area during SCC can be presented in the form of a table (Table 5):

Thus, during stress corrosion cracking, the corroded region has a distinct localized structure. The most active zone is located at the crack tip. where anodic dissolution of the metal occurs due to stress concentration, destruction of the passive film, and limited mass transfer. The crack channel acts as an occluded electrochemical cell: oxygen is rapidly depleted, metal cations and anions accumulate, hydrolysis of the dissolution products alters the pH and the resulting hydrogen can be adsorbed and penetrate the metal in the tip zone. Therefore, the speed and path of crack propagation are determined not by the composition of the bulk medium, but by the local chemistry, electrochemical potential, microstructure, and stress state immediately at the crack tip [47].

Thus, under cyclic loading, microcracks from the surface stress concentrators begin to form after a certain incubation period, which depends on the defect size (Table 6). It has been shown that the larger the concentrator, the faster the crack initiation occurs. The shortest incubation period was observed for the pits with diameters ranging from 0.9 μm to 1 mm. Once a microcrack has formed, its growth rate does not depend on the size of the concentrator.

Thus, the corrosive environment accelerates the crack initiation in pipeline steel. The time to the initiation of a microcrack from a surface stress concentrator in the corrosive environment is approximately two times shorter than in the air. The time of microcrack initiation depends on the size and shape of the surface stress concentrators. The cracks form more rapidly from pits with a high aspect ratio. In the corrosive environment with low hydrogen charging capacity (NS4 solution), cracks initiate from a surface concentrator with a diameter of 100–300 μm after 24–28 days (~300,000 cycles). The hydrogen concentration calculated using Equation (5) from the steady-state hydrogen penetration currents i_p,st_ (Table 4) generated in steel in this solution does not exceed 0.02 ppm at the corrosion potential of −600 mV (Table 4). In an environment with higher hydrogen charging capacity (CBS, pH 5.5), a higher concentration of dissolved hydrogen is found in the pipeline steel, namely, up to 0.19 ppm at the corrosion potential of −670 mV, and 0.4 ppm at high cathodic polarization potentials (Table 4). However, crack initiation is not significantly accelerated under these conditions (the incubation period is 25 days).

**Figure 6:**
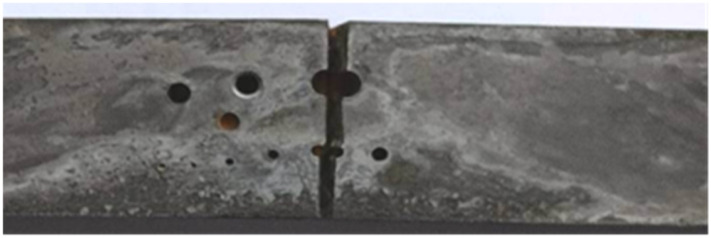
Crack initiation from a pit. Four-point bending method. Solution: CBS, pH 5.5 + 10 mM thiourea (CH_4_N_2_S). E = −1300 mV.

Under conditions of even more intense hydrogen charging of the pipeline steel, namely, in a medium with a hydrogen charging promoter (CBS, pH 5.5 + 10 mM thiourea) and at a high cathodic potential (−1300 mV), the hydrogen concentration in the steel reaches 0.98 ppm. Under these conditions, a crack initiates from a pit within 6 days. Initiation occurs from the largest pit (1000 μm) with the highest stress concentration around the pit (Figure 6).

Thus, the upper limit of safe hydrogen concentration in steel, in terms of crack initiation, can be estimated as 0.4 ppm, while pronounced stimulation of crack initiation occurs at concentrations above 0.98 ppm.

It should be noted that these data were obtained under conditions of high-intensity mechanical loading (up to ~σ_0.2_) and at a frequency of 0.15 Hz, which significantly exceed the loading regimes of gas pipelines. However, a quantitative assessment of the effect of the environment under conditions close to those of pipeline operation (loading up to 0.7 σT and extremely low frequency) can only be carried out in very prolonged experiments.

In addition to the characteristics of initiation and propagation at the early stages of SCC in pipeline steels, the possibilities for SCC inhibition were also studied. For this purpose, the specimen surfaces were modified with formulations based on organosilanes. After surface modification, the parameters of the resulting organosilicon surface layers were examined. In particular, the thicknesses of the resulting films were estimated from gravimetric measurements using Equation (2). The thicknesses of the surface layers calculated under the assumption of uniform surface coverage are presented in Table 6. As can be seen from Table 6, after modification of the alloy surface with a vinyl-containing silane, the thickness of the surface layer was about 356 nm. This value represents the average film thickness over the surface. Replacing vinylsilane with a mixture of vinyl- and aminosilanes led to a significant (more than twofold) increase in the layer thickness up to 0.884 μm (Table 6). In this case, the film is significantly thicker than that obtained with a vinyl-containing layer alone, which is likely due to a higher degree of condensation of silanol molecules (the product of reaction (5)) with hydroxyl groups on the metal surface (Equation (6)) and polymerization (polycondensation) of adjacent adsorbed silane molecules (Equation (7)), leading to the formation of a siloxane polymer.

(5) $$RSi(OR^{\prime })+3H_{2}O\to RSi(OH)+R^{\prime }OH$$

(6) $$R-\begin{array}{c} OH\\ |\\ Si\\ |\\ OH \end{array}-OH+HO-Me\begin{array}{c} -H_{2}O\\ \to \end{array}R-\begin{array}{c} OH\\ |\\ Si\\ |\\ OH \end{array}-O-Me$$

(7) $$OH-\begin{array}{c} R\\ |\\ Si\\ |\\ OMe \end{array}-OH+OH-\begin{array}{c} R\\ |\\ Si\\ |\\ OMe \end{array}-OH+OH-\begin{array}{c} R\\ |\\ Si\\ |\\ OMe \end{array}-OH\begin{array}{c} -H_{2}O\\ \to \end{array}OH-\begin{array}{c} R\\ |\\ Si\\ |\\ OMe \end{array}-O-\begin{array}{c} R\\ |\\ Si\\ |\\ OMe \end{array}-O-\begin{array}{c} R\\ |\\ Si\\ |\\ OMe \end{array}-OH$$

In the presence of an aminosilane, reactions (6) and (7) are expected to occur more readily, as primary amines are known to act as catalysts for these processes, including acting as autocatalysts [55]. The silanol groups of adjacent molecules condense (in accordance with Equation (7)), polymerizing and forming linear polymeric siloxane chains oriented not only parallel to the surface but also in transverse directions, thereby increasing both the degree of crosslinking and the thickness of the polymer layer (where polycondensation occurs in the direction perpendicular to the surface). Thus, in this case, three-dimensional growth of the film occurs (not only horizontally—along the surface, but also vertically—perpendicular to the surface), which is responsible for the increase in the film thickness.

The use of modifying formulations based on the mixtures of vinylsilane with corrosion inhibitors, BTA, and CAB, leads to the formation of significantly thinner films: they are nearly an order of magnitude thinner than those after the surface modification with a VS solution, and more than an order of magnitude thinner compared to the [VS + AS] mixture. In fact, for the [VS + BTA] and [VS + CAB] mixtures, the film thickness is 34.7 and 29 nm, respectively (Table 6). This is apparently due to the incomplete polycondensation reaction of silane (silanol) molecules (Equation (7)) due to the lower catalytic activity of BTA and catamine AB that contain no primary amino groups. Most likely, in these cases polymerization occurs predominantly on the surface, resulting in polymer chains oriented mainly parallel to the surface rather than perpendicular to it, which would otherwise promote an increase in the layer thickness. In addition, amino groups (not primary groups but those present in BTA and catamine AB molecules) can react with silanol groups (involved in condensation and polycondensation reactions) (Equation (8)), reducing their total amount and thereby decreasing the extent of the polycondensation reaction of adsorbed silane/silanol molecules and, consequently, the degree of polycondensation of the azole- and amine-containing siloxane surface layer (compared to the vinylsilane layer). This may explain the smaller thicknesses of the layers formed upon surface modification with [VS + BTA] and [VS + KAB] mixtures compared to modification with the VS solution (Table 7). However, in these cases it can be expected that the layers formed, despite their smaller thickness, will be more densely cross-linked via additional silazane Si–N= bonds (compared to silane layers that do not contain amino groups) formed by reaction (8). Thus, surface modification with organosilane mixtures containing amine-bearing organic corrosion inhibitors is expected to produce thin (nanoscale, see Table 6) but densely cross-linked polymer-oligomer films with low permeability (due to the high degree of crosslinking) to electrolyte components.

(8) $$R-\begin{array}{c} |\\ Si\\ | \end{array}-OH+NH-R^{\prime }\begin{array}{c} -H_{2}O\\ \to \end{array}R-\begin{array}{c} |\\ Si\\ | \end{array}-\begin{array}{c} |\\ N \end{array}-R^{\prime }$$

**Figure 7:**
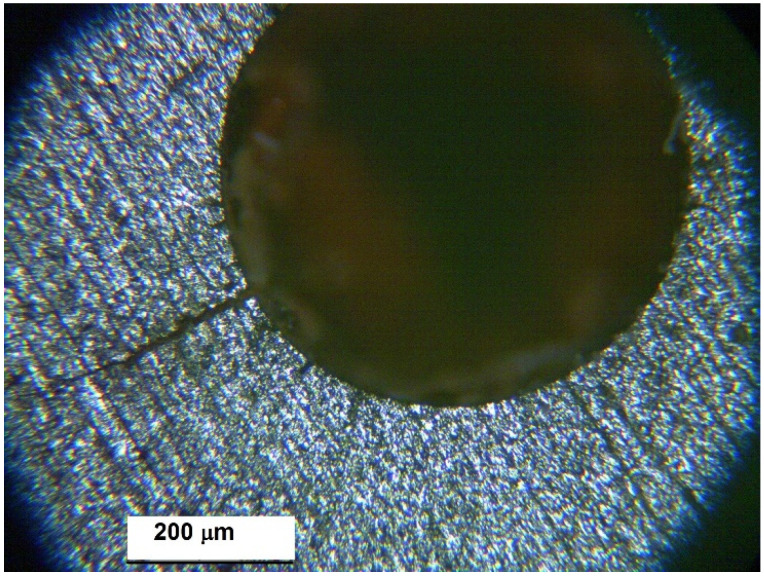
Crack initiation on the specimen modified with a 1 wt. % aqueous solution of VS with a hole as the defect initiator (d = 1 mm), after 28 days of testing. Four-point bending method. Solution: NS4.

**Figure 8:**
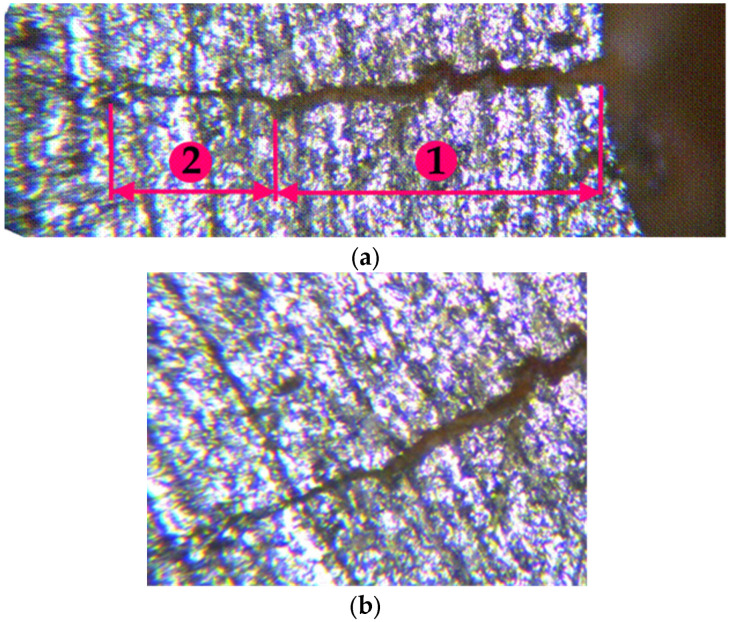
Appearance of the crack on the specimen modified with a VS solution with a hole as the defect initiator: (**a**) The entire crack (area 1 and 2) after 28 days of corrosion-mechanical testing. Four-point bending method. Solution: NS4; (**b**) Enlarged (×20) image of the crack in area 2 in (**a**).

It was found that the formation of organosilicon layers on the steel after modification with organosilane formulations inhibits the development of stress corrosion cracking (Table 6). Even the least effective vinylsiloxane surface layer (modification with VS solution), which does not reduce but even increases the crack growth rate at the initial stage of its development, increases the incubation period of crack initiation almost six-fold. The crack (Figure 7) appeared on the specimen surface after 28 days of testing (for unmodified steel—after 5 days and had the following dimensions: the mouth width was 20 μm, while the length after 28 days of testing was 480 μm (wide part—320 μm, region 1, Figure 8; narrow part—160 μm, region 2, Figure 8).

A more detailed study of the growing crack showed (Figure 8b) that, as expected on the basis of literature data [56], under our conditions (i.e., at near-neutral pH), the nature of crack growth is transcrystalline (rather than intercrystalline). As noted above, the cracks in the unmodified specimens initiated after 24–28 days only from natural stress concentrators (pits). In the specimens with the artificial defect initiators in the form of drilled holes, the crack formation occurred significantly earlier, i.e., after 5–7 days of testing (Table 6). Thus, the vinylsiloxane surface layer increases the incubation period of corrosion crack initiation; however, no decrease in the crack growth rate was observed. Moreover, a slight increase (by ~13%) in the crack growth rate was observed. This can be explained as follows: upon crack formation, the surface siloxane film is destroyed and remains on the “external” (relative to the crack) metal surface, without penetrating inside the crack and therefore not affecting its propagation rate. In the case of an incomplete polycondensation reaction, the unreacted vinylsilane molecules could remain on the surface; these molecules can desorb and, upon entering the solution, migrate inside the crack toward its tip, adsorb on the metal surface at the crack tip, and affect its propagation, for example, by enhancing steel passivation [57] and by reducing the crack growth rate. Apparently, the polycondensation reaction of vinylsilanol occurs to rather a high extent, and the amount of unreacted silane (silanol) molecules is insufficient to passivate the metal at the crack mouth. This is especially true, considering that the number of vinylsilanol molecules not involved in reactions (6) and (7) is reduced due to adsorption on the crack walls, which does not affect the crack growth.

**Figure 9:**
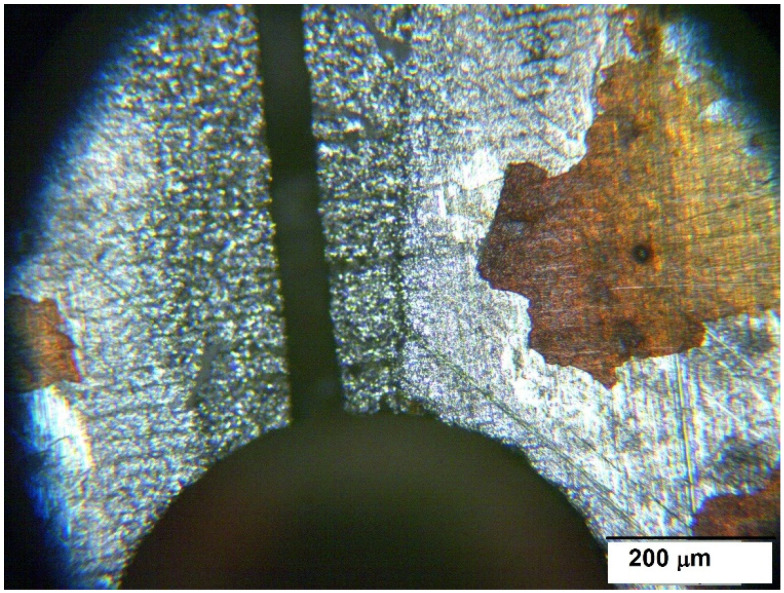
Appearance of the crack on the specimen modified with a [VS + AS] mixture with a hole defect initiator, after 22 days of corrosion-mechanical testing. Four-point bending method. Solution: NS4.

The use of a mixture of vinyl- and aminosilanes for the surface modification of the steel led to the crack initiation after 22 days of testing in the NS4 solution (Figure 9).

The corrosion crack had the following dimensions: the crack mouth width was about 110 μm and the crack length was 7.5–8 mm. The crack growth rate at the initial stage of its propagation was nearly 3.5 times smaller than that in the unmodified steel. Apparently, in this case, the amount of unreacted silane molecules is sufficient for desorption from the metal surface, migration inside the crack toward its tip, adsorption on the metal surface at the crack tip, and enhancement of metal passivation in this region.

Thus, the mixed vinyl- and amine-containing siloxane layer inhibits both the initiation and propagation of a “new” corrosion crack in pipeline steel more effectively than the vinylsiloxane layer alone.

It is known [57] that mixtures of organosilanes with organic corrosion inhibitors are significantly more effective in mitigating metal corrosion than the individual components of such mixtures. Therefore, mixtures of organosilanes with corrosion inhibitors were used for surface modification of pipeline steel: a mixture of VS with catamine AB (CAB), an organic amine-type corrosion inhibitor [58], and a mixture of VS with 1,2,3-benzotriazole (BTA), an organic azole-type corrosion inhibitor [59].

**Figure 10:**
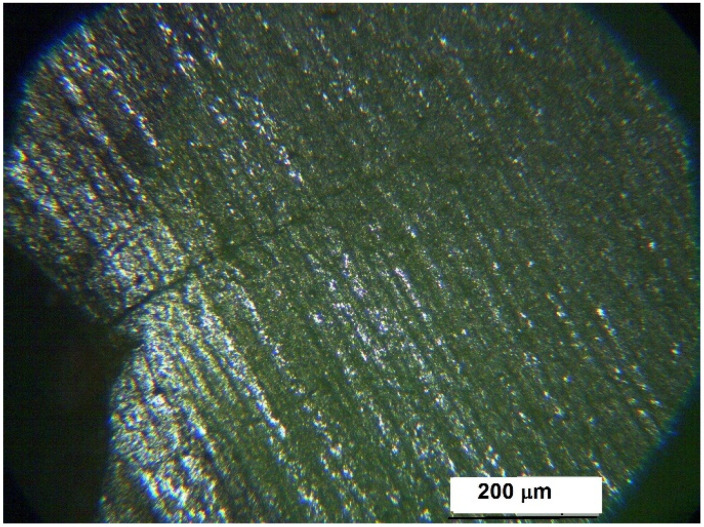
Appearance of the crack on the specimen modified with a [VS + CAB] mixture with a hole as the defect initiator, after 23 days of testing. Four-point bending method. Solution: NS4.

It was found that after the surface modification of the pipeline steel with the [VS + CAB] mixture, a crack in a specimen under cyclic loading in the four-point bending scheme in NS4 solution appeared after 23 days of testing (Figure 10). The crack mouth width did not exceed 25 μm, while the crack length was 40 μm. The crack growth rate was 2.01 × 10^−8^ mm/s, i.e., 7.2 times smaller than that in the unmodified steel. Thus, the surface layer formed upon modification with vinylsilane and catamine AB demonstrated inhibiting properties towards crack formation in the pipeline steel, although it was slightly less effective than the vinylsiloxane layer.

**Figure 11:**
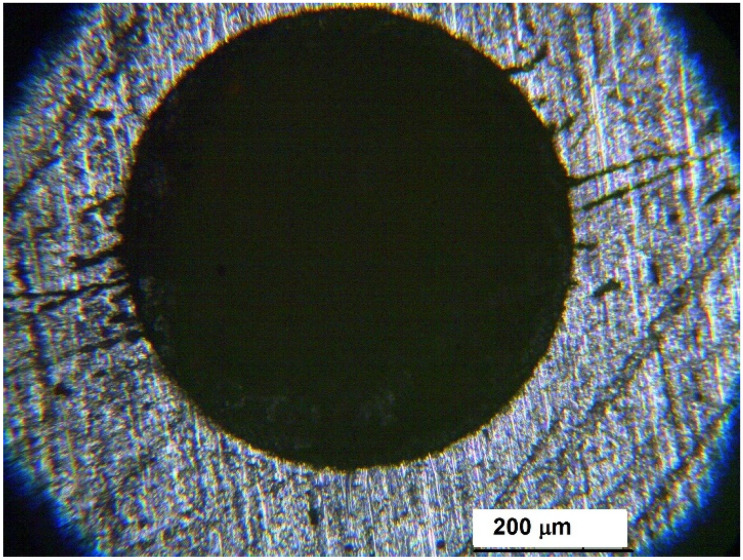
Appearance of the crack on the specimen modified with a [VS + BTA] mixture with a hole as the defect initiator, after 36 days of testing. Four-point bending method. Solution: NS4 + borate buffer (pH 6.7).

**Figure 12:**
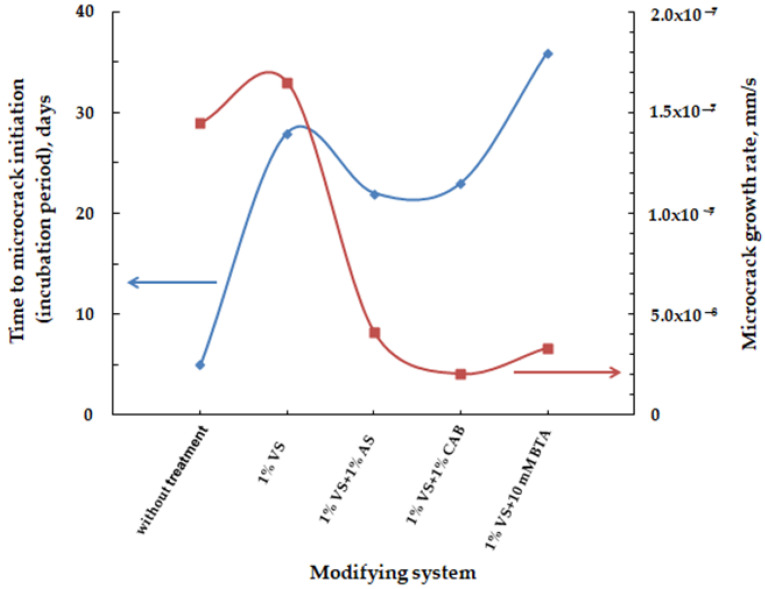
Effect of organosilicon surface layers on the stress corrosion cracking (SCC) of X70 pipeline steel. The cracks initiated from an “artificial” stress concentrator with a diameter of 1 mm. Four-point bending method. Solution: NS4 + borate buffer (pH 6.7). The error in calculating the crack growth rate is 1 × 10^−9^ mm/s. Thus, it can be expected that surface modification of the steel with formulations based on vinylsilane will result in inhibition of corrosion crack initiation in stressed pipeline steels. The most effective inhibition, evaluated by both the incubation period and crack growth rate, was achieved with the surface siloxane-azole layer formed upon surface modification with a [VS + BTA] mixture. The modifying formulations under study can be ranked in the following order of their inhibiting efficiency: Steel (St) < (St + [VS + AS]) < (St + [VS + CAB]) < (St + VS) < (St + [VS + BTA]).

Upon surface modification of the steel with a [VS + BTA] mixture, crack initiation (Figure 11) occurred after a longer induction period compared to the other modifiers, namely, in 36 days. The incubation period of crack initiation in this case was more than seven times longer than that for the unmodified steel. The crack had the following dimensions: the crack mouth width was 10–15 μm, while the crack length was 250–280 μm. The crack growth rate at the initial stage of propagation was 3.31 × 10^−8^ mm/s, i.e., 4.6 times smaller than in the unmodified steel (Figure 12). In this case, the following mechanism of crack growth inhibition can be proposed. In addition to the siloxane-azole polymer layer on the metal surface responsible for the incubation period of crack initiation, unreacted BTA molecules are present, which, as noted above, can desorb from the steel surface into the solution bulk and penetrate into the opened crack. By adsorbing on the freshly formed metal surface at the crack tip, BTA promotes steel passivity [60], thereby suppressing crack growth. It is likely that a similar mechanism is valid for the mixture of vinylsilane with another corrosion inhibitor, catamine AB (see above). The surface polymeric siloxane-azole/amine layer acts as some kind of reservoir for organic corrosion inhibitors, which also include unreacted silane molecules. This reservoir begins to function as a source of corrosion inhibitors from the moment when the modified surface comes into contact with a solution.

Thereby it was found that the formation of organosilicon layers on the steel after modification with organosilane formulations inhibits the development of stress corrosion cracking. The data on crack growth rates and incubation periods of its occurrence for the studied modifying compositions have been summarized and presented in Figure 12. It can be seen that even the least effective vinylsiloxane surface layer (modification with VS solution), which does not reduce but even increases the crack growth rate at the initial stage of its development (Figure 12), increases the incubation period of crack initiation almost six-fold (Figure 12). The crack (Figure 7) appeared on the specimen surface after 28 days of testing (for unmodified steel–after 5 days (Figure 12)).

## 4. Conclusions

It has been shown that in a corrosive environment with low hydrogen charging capacity (NS4 solution), cracks in a specimen with stress concentrators in the form of “natural” pits appear after 24–28 days (~300,000 cycles). The hydrogen concentration generated in the steel in this solution does not exceed 0.31 ppm. Crack growth in the corrosive environment and in the air occurred at a rate of 7–13 μm/day for Mannesmann steel and 10–15 μm/day for KhTZ steel.

In tests of specimens with holes as the defect initiators, the crack formation occurred earlier than with the “natural” pit initiators. The cracks appeared after 5–7 days of testing from the edges of holes with diameters ranging from 600 μm to 1 mm.

In the environment with higher hydrogen charging capacity (CBS, pH 5.5; C_H_ ~ 0.75 ppm), the crack initiation at the corrosion potential did not accelerate considerably: the incubation period was 25 days.

The upper limit of safe hydrogen concentration in steel, in terms of crack initiation, is 0.75 ppm, while significant acceleration of crack initiation occurs at hydrogen concentrations above 3 ppm.

It has been found that surface modification of the steel with formulations based on vinylsilane leads to the inhibition of corrosion crack initiation in stressed pipeline steels. The modifying formulations can be ranked in the following order of their inhibiting efficiency:

Steel (St) < (St + [VS + AS]) < (St + [VS + CAB]) < (St + VS) < (St + [VS + BTA])

## Figures and Tables

**Table 1:** Chemical composition of steel specimens manufactured by KhTZ and Mannesmann.

Steel	C	Si	Mn	P	S	Cr	Ni	Cu	Al	Ti	V	Nb
**KhTZ**	0.115	0.34	1.63	0.021	0.003	0.04	0.02	0.007	0.030	0.07	-	-
**Mannesmann**	0.08	0.48	1.63	0.023	0.003	0.024	0.013	0.008	0.033	-	0.076	0.027

**Table 2:** Composition of NS_4_ model test solution (g/L) determined from field studies [34].

Compound	Concentration, g/L
**KCl**	0.122
**NaHCO_3_**	0.483
**CaCl_2_ × 2H_2_O**	0.181
**MgSO_4_ × 7H_2_O**	0.131

**Table 3:** Compositions of organosilicon solutions used for surface modification of the specimens.

No.	Composition of Modifying Solution		
	Organosilane	Organic Corrosion Inhibitors	Solvent
1	1 wt.% solution of VS *	–	water
2	1 wt.% solution of VS * + 1 wt.% solution of AS **	–	water
3	1 wt.% solution of VS *	0.12% BTA ***	ethanol
4	1 wt.% solution of VS *	1% CAB ****	water

* VS—vinyltrimethoxysilane CH_2_ = CHSi(OCH_3_) (produced by Silan LLC, Moscow city, Russia); ** AS—γ-aminopropyltriethoxysilane NH_2_-(CH_2_)_3_Si(OC_2_H_5_)_3_ (produced by Silan LLC, Moscow city, Russia); *** BTA—organic corrosion inhibitor of the azole class, 1,2,3-benzotriazole C_6_H_5_N_3_. (produced by Khimstar LLC, Volzhsky city, Volgograd region, Russia); **** CAB—organic corrosion inhibitor catamine AB, which is a mixture of alkylbenzyldimethylammonium chlorides of general formula [C*_n_*H_2*n*+1_-C_6_H_4_-*N*^+^(CH_2_)_3_]Cl^−^, where *n* is the alkyl chain length, *n* = 12–18 (produced by Neokhimax LLC, Moscow region, Domodedovo city, Russia).

**Table 4:** Steady-state currents of hydrogen penetration through the steel membrane and calculated hydrogen concentrations in the near-surface layer of X70 steel. The measurement error of the potential is 0.1 mV, and measurement error of the current density is 0.01 μA/cm^2^.

Test Environment	Potential, mV (vs. Silver Chloride Electrode)	Steady-State Current of Hydrogen Penetration i_p,st_, μA/cm^2^	Concentration of Hydrogen in Pipeline Steel in the Crack Initiation Region, C_H_, ppmw
**NS4 with BBS**	−600	0.37	0.02
**CBS**	−670	–	0.19
**CBS**	−1600	12.4	0.40
**CBS**	−1700	12.7	0.40
**CBS + 10 mM thiourea**	−1300	30.9	0.98

**Table 5:** Structure of the corroded area of metal during SCC.

Zone	Role
External surface/crack mouth	Contact with a bulk medium: often a cathodic region: the crack may be thin and branched
Crack walls	Partially passivated/covered with corrosion products: limits mass transfer
Electrolyte inside the crack	Occluded environment: low oxygen, different pH, increased ion concentrations
The top of the crack	Main active anode zone: metal dissolution, filmrupture/recovery
Pre-crack tip zone	Mechanical process zone: plastic deformation, hydrogen, dislocations, grain boundaries, traps
Corrosion products/oxide-hydroxide film	They can lock the environment, change pH and ion transport, and influence the introduction of hydrogen

**Table 6:** Initiation time and growth rate of microcracks on the surface of KhTZ and Mannesmann steels as a function of the size of the initial stress concentrator (pit) in the corrosive environment and in the air.

Parameter	KhTZ Steel			Mannesmann Steel		
**Diameter of the initial pit, μm**	300	600	900–1000	100	600	900
**Aspect ratio of the initial pit, diameter: depth ratio**	1:1	1:2	1:2.5	1:1	1:2	1:2.5
**Time to microcrack initiation in the air, days**	55	15	9	60	16	9
**Time to microcrack initiation in NS4 environment, days**	24	7	5	28	9	6
**Microcrack growth rate after the initiation from the pit in the air**	(1.1–1.7) × 10^−7^ mm/s			(0.8–1.5) × 10^−7^ mm/s		
**Microcrack growth rate after the initiation from the pit in NS4 environment**	(1.1–1.7) × 10^−7^ mm/s			(0.8–1.5) × 10^−7^ mm/s		

**Table 7:** Thicknesses of films formed on the surface of X70 pipeline steel after modification with organosilane-based formulations.

System	Layer Thickness, μm
**Non-modified steel (St)**	0
**St + VS**	0.356
**St + (VS + AS)**	0.884
**St + (VS + BTA)**	0.035
**St + (VS + CAB)**	0.029

## Data Availability

The original contributions presented in this study are included in the article. Further inquiries can be directed to the corresponding author.
